# Modern Sociology

**Published:** 1902-09-20

**Authors:** 


### Modern Sociology.

### CHILD-HAWKERS.

Miss Rosa M. Barrett, whose studies of child-life among the less favoured classes we have noticed before, has been investigating the condition of those children who earn a living by selling in the street, and has tried to trace their subsequent careers. It is almost unnecessary to say that she considers the custom of sending children to vend their wares in the street a very serious social danger. No arguments are needed to prove that street-hawking is a bad thing for girls; the attendant moral dangers are obvious. But it is a bad thing even for boys, though it may not be practicable to forbid it entirely. The comparative freedom of the life, the loafing, the absence of any set work, the irregular hours, all tend to make the boy who sells on the street a vagrant and a lounger for the rest of his life. The habit of casual living and chance earning, begun in youth, is only too apt to continue throughout maturer life. When once these vagrant habits have been learned it is, says Miss Barrett, "impossible, or at least most difficult, to bring the child under restraint, or to teach it habits of industry. . . . In France it has been found that this class of children, and the children of vagrants, are more demoralised than juvenile criminals—are the most difficult to deal with or to reform."

Perhaps the mere fact of the early independence which these children obtain is not the only reason why, having once begun, they go on as vagrants. Street-selling, whether carried on for the whole day or only before and after school, interferes greatly with education. The children are playing truant as much as they dare, and if the fear of the law makes their parents send them to school, they are, if they have been working before and after school hours, quite unfit to learn. They grow up, as a rule, with neither manual nor mental training. Therefore, even if they desire to live honestly when they grow up, they are unable to make any but the poorest living. "When the boys get big and want to earn more," says Miss Barrett, "they are too old to learn a trade, and they increase that difficult class, of next to no value in the labour market, living always upon the verge of starvation, our unskilled labourers. . . . A skilled industrious workman is almost certain of regular work at good wages, but the supply of unskilled labourers far exceeds the demand." It is from this class that the ranks of paupers and criminals are largely recruited. The criminal class is, speaking generally, the uneducated class. In Great Britain only about 3 per cent. of the prisoners are well educated, in Ireland more than half are wholly or almost wholly illiterate. Everywhere, Miss Barrett declares, unskilled labourers form the bulk of the prisoners in our gaols—about four-fifths of the whole. And many of these are young people. A third of all the burglars are under 21 in England, and a fourth of all convictions for larceny are against juveniles under 16, and a large proportion of these, it is stated, come from street-hawkers. They also contribute largely to the inmates of the reformatory and industrial schools. In Manchester in one year, they—that is, street-hawkers—formed 67 per cent., and in Leeds 60 per cent., of those committed to these schools.

These are the facts in our own country, where there is practically no restriction on the employment of young children as street-hawkers, for the restrictions placed indirectly in the way of the business by the Education Act are habitually evaded. Other countries have ventured to deal with a custom which is admittedly full of danger. It is worth while to inquire with what results. In the State of New York, since 1896, girls have been forbidden to sell in the streets at night, and the same law applies even to boys, if they are under sixteen years of age. Parents who permit boys and girls to hawk in the streets are regarded as being guilty of a misdemeanour, and if they do not restrain the children the latter may be removed to an asylum. Since that law has been passed there has been a decrease of 50 per cent. in juvenile crime, and among girls the decrease in criminals, waifs, and vagrants has been even greater. Other States do the same as New York. All children under sixteen years of age are forbidden to beg or sell things on the road in California, and peddling is forbidden to children under fourteen in Illinois, Minnesota, Kansas, Rhode Island and the other New England States, while in Massachusetts no minor who cannot read and write may be employed thus without a special permit from the school commissioners. In Maryland, not only are the children themselves forbidden to sell, but children under eight years of age are not even permitted to accompany adults who are begging or peddling. In Canada news-girls are unknown, and news-boys must be licensed, wear a badge, and attend school. In others of our colonies—Victoria and South Australia—children under fifteen are not allowed to sell in the streets unless they are registered and have passed a certain school standard. Any child selling without this permission may be apprehended and made a ward of the State. In Prussia, children under fourteen are forbidden to sell in the streets, and are not even permitted to deliver goods between the hours of 7 P.M. and 7 A.M. Thus in our own colonies and in several other countries it is clearly held that the street is the highway to crime, and that if the mischief is to be nipped in the bud, children must not be allowed to hang about the streets with a view to earning their living.

Doubtless this will seem a hardship to many, and the familiar story, so popular in magazines of a certain class, of the sick or widowed mother, supported by the child who peddles papers or toys in the street, will be brought up. But as a matter of fact, this case is but a rare one. The majority of the children who are sent out to vend papers or trifles in the streets of our cities have parents—idle, dissolute, thriftless parents, who are thereby teaching their children to grow up like themselves. Consideration for them should certainly not induce either the State or private charity to consent to the children following a manner of trade which is more than likely to prove demoralising. Miss Barrett is clearly of opinion that the education laws should be strictly enforced, and truancy punished with the utmost severity. If parents can be compelled to fulfil their duties so much the better, but if not, then the State should step in, and take the matter in its own hand. It may be protested that this step would please no one better than the parents who have so little regard for their responsibilities. They would be only too glad to get their children taken off their hands. But this point of view would be altered if they were compelled, as they should be, to contribute a reasonable proportion of the sum required for the maintenance of a child in an institution. At present, in England, a parent pays one shilling a week for every child he has in an industrial school. In Australia, parents who are compelled thus to delegate their responsibilities pay on an average a twelfth of their earnings for each child thus removed from their control. It cannot be doubted that an addition to the expense of keeping a child in an industrial school would have a very quickening influence on parental responsibility, but to make this lever effective in saving the children it would need to be combined with stricter laws regarding the kind and the amount of work which children were permitted to undertake, and the hours of the day during which they were permitted to work at all.

